# Epidemiology and Prevalence of Dyslipidemia Among Adult Population of Tehran: The Tehran Cohort Study

**DOI:** 10.34172/aim.2024.10

**Published:** 2024-02-01

**Authors:** Akbar Shafiee, Sina Kazemian, Arash Jalali, Farshid Alaeddini, Soheil Saadat, Farzad Masoudkabir, Hamed Tavolinejad, Ali Vasheghani-Farahani, Vicente Artola Arita, Saeed Sadeghian, Mohamamdali Boroumand, Abbasali Karimi, Oscar H Franco

**Affiliations:** ^1^Tehran Heart Center, Cardiovascular Diseases Research Institute, Tehran University of Medical Sciences, Tehran, Iran; ^2^Cardiac Primary Prevention Research Center, Cardiovascular Diseases Research Institute, Tehran University of Medical Sciences, Tehran, Iran; ^3^Department of Epidemiology and Biostatistics, School of Public Health, Tehran University of Medical Sciences, Tehran, Iran; ^4^Department of Emergency Medicine, University of California, Irvine, California, USA; ^5^Julius Center for Health Sciences and Primary Care, University Medical Center Utrecht, Utrecht, the Netherlands

**Keywords:** Dyslipidemia, Epidemiology, Hypercholesterolemia, Hypertriglyceridemia, Prevalence

## Abstract

**Background:**

Dyslipidemia is among the leading risk factors for cardiovascular diseases (CVDs), with an increasing global burden, especially in developing countries. We investigated the prevalence of dyslipidemia and abnormal lipid profiles in Tehran.

**Methods:**

We used data from 8072 individuals aged≥35 from the Tehran Cohort Study (TeCS) recruitment phase. Fasting serum total cholesterol (TC), low-density lipoprotein-cholesterol (LDL-C), high-density lipoprotein-cholesterol (HDL-C), and triglyceride were measured. Dyslipidemia was defined according to the National Cholesterol Education Program Adult Treatment Panel III criteria, and high LDL/HDL was defined as a ratio>2.5. The age-sex standardized prevalence rates were calculated based on the 2016 national census. Furthermore, the geographical distribution of dyslipidemia and lipid abnormalities was investigated across Tehran’s zip code districts.

**Results:**

The age-sex standardized prevalence was 82.7% (95% CI: 80.1%, 85.0%) for dyslipidemia, 36.9% (95% CI: 33.8%, 40.1%) for hypertriglyceridemia, 22.5% (95% CI: 19.9%, 25.4%) for hypercholesterolemia, 29.0% (95% CI: 26.1%, 32.1%) for high LDL-C, 55.9% (95% CI: 52.6%, 59.2%) for low HDL-C, and 54.1% (95% CI: 50.9%, 57.3%) for high LDL/HDL ratio in the Tehran adult population. The prevalence of dyslipidemia, low HDL-C, and high LDL/HDL ratio was higher in the northern regions, hypercholesterolemia was higher in the southern half, and high LDL-C was more prevalent in the middle-northern and southern areas of Tehran.

**Conclusion:**

We found a high prevalence of dyslipidemia, mainly high LDL/HDL in the Tehran adult population. This dyslipidemia profiling provides important information for public health policy to improve preventive interventions and reduce dyslipidemiarelated morbidity and mortality in the future.

## Introduction

 Cardiovascular diseases (CVDs) represent the primary cause of mortality and disease burden worldwide.^1^ The prevalence of CVDs has nearly doubled since 1990 and reached 523 million cases in 2019; a steady increase in the mortality rate has been observed throughout these years.^1^ Dyslipidemia is among the leading risk factors for CVDs that has been tightly knotted with atherosclerosis pathophysiology and plaque formation, known as the leading underlying cause of CVDs.^2-4^ The global burden of dyslipidemia showed an increasing trend due to the aging population, inadequate physical activity, behavioral risk factors, and obesity, particularly in developing countries.^1,5^ In 2015, Iran was the third most affected country in the Eastern-Mediterranean region with 54.1% prevalence of hypercholesterolemia and recorded 46% of CVD deaths according to the World Health Organization (WHO) estimates.^6^

 Tehran is the capital and the most populated city of Iran; it is also recognized as the third largest metropolitan area in the Eastern Mediterranean region, with more than 9 million population and significant ethnic diversity.^7^ A nationwide survey in 2021 found that about 81% of the Iranian adult population had at least one lipid abnormality, and several studies have reported different rates of dyslipidemia across various regions of the country.^8-11^ Previous epidemiological studies in Tehran were limited by district-level samplings, and small sample sizes, with uncertainties regarding the current prevalence of dyslipidemia rates in Tehran.^12-14^

 Nevertheless, the current understanding of dyslipidemia prevalence in Tehran remains limited, particularly across its diverse districts and among different age and sex groups. It is essential to highlight that recognizing the epidemiology of dyslipidemia as a modifiable cardiovascular risk factor could lead to more effective prioritization of interventions. Such targeted strategies, based on regional profiling, hold significant potential for controlling and preventing CVDs. In this study, we aim to investigate the prevalence of dyslipidemia, its geographic distribution and associated risk factors among participants of the Tehran Cohort Study (TeCS).

## Materials and Methods

###  Study Design and Participants

 In this study, we used the TeCS recruitment data, an ongoing prospective population-based cohort study of adult citizens of Tehran aged 35 and above. Details of the TeCS design and sampling have been previously published.^15^ In summary, a total of 9,170 adults aged ≥ 35 years were selected from 4215 households using a systematic random sampling method based on their residential zip codes to represent all districts of Tehran. We invited all the participating individuals for an interview and initial evaluation at the Tehran Heart Center, and 8296 individuals underwent assessment from May 2016 to February 2019. For this analysis, we excluded 224 patients who lacked information on dyslipidemia history. Furthermore, 122 participants who underwent non-fasting laboratory sampling were excluded from the analysis of lipid test results.

###  Data Collection and Measurements

 We interviewed every participant using a designated comprehensive checklist on demographic characteristics, drug, and past medical history, family history of coronary artery disease (CAD), smoking, and drinking habits. In addition, we used physical activity questions from the STEPs instrument version 3.2 to measure the participants’ physical activity levels.^16^ All participants underwent standard anthropometric evaluation, including body height, weight, waist, and hip circumference measurements by a trained nurse. Besides, blood pressure was measured on the left arm in a standard setting by a trained nurse using a digital sphygmomanometer (M6 Comfort Omron, Omron Healthcare, Kyoto, Japan).^17^ If the first recording was above 140/90 mm Hg, we performed a subsequent measurement on the same arm following a five-minute rest period. Afterward, a venous blood sample was obtained from every individual following a 12-hour overnight fast to check fasting plasma glucose (FPG), creatinine, total cholesterol (TC), low-density lipoprotein-cholesterol (LDL-C), high-density lipoprotein-cholesterol (HDL-C), and triglyceride. The biochemistry measurements were performed by the experienced laboratory staff at Tehran Heart Center using Roche kits (Roche Diagnostics, Basel, Switzerland) and COBAS Integra 400 plus device (Roche Diagnostics, Basel, Switzerland).

###  Definitions

 Dyslipidemia was defined based on laboratory findings, previous diagnosis, or self-reported use of lipid-lowering medications, in accordance with the Third Report of the National Cholesterol Education Program, Expert Panel on Detection, Evaluation, and Treatment of High Blood Cholesterol in Adult Treatment Panel III.^18^ Individuals with either one or a combination of hypercholesterolemia, high LDL-C, low HDL-C, and hypertriglyceridemia were considered to have dyslipidemia. Hypercholesterolemia was characterized as TC concentrations of ≥ 200 mg/dL (≥ 5.2 mmol/L), high LDL-C defined as LDL-C ≥ 130 mg/dL (≥ 3.4 mmol/L), low HDL-C defined as HDL-C < 40 mg/dL (< 1.03 mmol/L) in men, and < 50 mg/dL (< 1.29 mmol/L) in women, hypertriglyceridemia was characterized as triglyceride ≥ 150 mg/dL (≥ 1.7 mmol/L), and high LDL/HDL as ratio > 2.5.^18,19^

 Hypertension was defined as increased systolic blood pressure (SBP) ≥ 140 mm Hg or diastolic blood pressure (DBP) ≥ 90 mm Hg), previous hypertension diagnosis, or antihypertensive treatment.^20^ Diabetes mellitus was described as a self-report of previous diabetes mellitus diagnosis or treatment with oral antidiabetic agents, insulin, or FPG ≥ 126 mg/dL (7.0 mmol/L) after 8-12 hours of overnight fasting. Cerebrovascular disease was determined based on a prior history of stroke or transient ischemic attack. We calculated the mean arterial pressure (MAP) using the following equation: (SBP + 2*DBP)/3.

###  Statistical Analysis

 In this study, categorical variables were presented as numbers (percentages) and compared for differences between groups using the chi-square test. Continuous variables were presented as mean ± standard deviation and assessed between the groups using either an independent *t* test or one-way analysis of variance (ANOVA) test, as appropriate. Furthermore, skew-distributed variables were reported as median with interquartile range boundaries and compared using the Mann–Whitney U test. The age-sex standardized prevalence of dyslipidemia and abnormal lipid profile were estimated in both men and women considering their age distribution, consistent with the 2016 national census, and was described with a 95% confidence interval (CI). Furthermore, we illustrated the prevalence of dyslipidemia and impaired lipid profile in different zip code regions on the Tehran map using *shp2dta* and *spmap* modules that visualize spatial data from every region onto the map in the Stata statistical software, version 14.2. Statistical analyses were performed by SPSS Statistics for Windows, version 23.0 (Armonk, NY: IBM Corp.). A two-sided *P* value < 0.05 was considered statistically significant.

## Results

 This study analyzed data from 8072 individuals (97.3% of total TeCS participants). The mean age was 53.8 ± 12.71, and 4375 (54.2%) were women. The mean body mass index (BMI) was 28.0 ± 4.8 kg/m^2^, while 5762 (72.1%) and 2426 (30.3%) were in the overweight and obese range, respectively. The most common comorbidities were dyslipidemia, hypertension, and diabetes mellitus. The baseline characteristics of the study participants are reported in (Table 1).

**Table 1:** Baseline Characteristics of Participants and Comparison Between the Individuals With and Without Dyslipidemia

**Characteristics**		**Total (N=8072)**	**Dyslipidemia**^a^ ** (n=6716)**	**Non-dyslipidemia (n=1356)**	***P*** **value**^b^
Age, year		53.8 ± 12.7	54.3 ± 12.5	51.3 ± 13.2	< 0.001
Sex, n (%)	Women	4375 (54.2)	3635 (54.1)	740 (54.6)	0.763
	Men	3697 (45.8)	3081 (45.9)	616 (45.4)	
BMI, kg/m^2^		28.0 ± 4.8	28.4 ± 4.7	26.1 ± 4.9	< 0.001
BMI Subgroups, n (%)	< 20	218/7996 (2.7)	100/6649 (1.5)	118/1347 (8.8)	< 0.001
	20-24.9	2016/7996 (25.2)	1521/6649 (22.9)	495/1347 (36.7)	
	25-29.9	3336/7996 (41.7)	2867/6649 (43.1)	469/1347 (34.8)	
	30-34.5	1761/7996 (22.0)	1560/6649 (23.5)	201/1347 (14.9)	
	≥ 35	665/7996 (8.3)	601/6649 (9.0)	64/1347 (4.8)	
Waist-to-hip ratio		0.92 ± 0.07	0.92 ± 0.07	0.89 ± 0.08	< 0.001
Education years, n (%)	Illiterate	568/8053 (7.1)	510/6699 (7.6)	58/1354 (4.3)	< 0.001
	1-5	821/8053 (10.2)	712/6699 (10.6)	109/1354 (8.1)	
	6-12	4194/8053 (52.1)	3499/6699 (52.2)	695/1354 (51.3)	
	> 12	2470/8053 (30.7)	1978/6699 (29.5)	492/1354 (36.3)	
Diabetes mellitus, n (%)		1491/8025 (18.6)	1384/6669 (20.8)	107/1356 (7.9)	< 0.001
Hypertension, n (%)		2291/8057 (28.4)	2087/6701 (31.1)	204/1356 (15.0)	< 0.001
Coronary heart disease, n (%)		767/8058 (9.5)	716/6703 (10.7)	51/1355 (3.8)	< 0.001
Cerebrovascular disease, n (%)		118/8057 (1.5)	107/6702 (1.6)	11/1355 (0.8)	0.029
Chronic kidney disease, n (%)		70/8072 (0.9)	66/6716 (1.0)	4/1356 (0.3)	0.013
Statin, n (%)		1757/8053 (21.8)	1757/6699 (26.2)	0 (0.0%)	< 0.001
Non-statin lipid-lowering agents, n (%)		162/8053 (2.0)	162/6699 (2.4)	0 (0.0%)	< 0.001
Family history of CAD, n (%)		758/8072 (9.4)	640/6716 (9.5)	118/1356 (8.7)	0.341
Tobacco, n (%)	Current	1551/8054 (19.3)	1265/6700 (18.9)	286/1354 (21.1)	0.030
	Former	323/8054 (4.0)	282/6700 (4.2)	41/1354 (3.0)	
	Never	6180/8054 (76.7)	5153/6700 (76.9)	1027/1354 (75.8)	
Alcohol, n (%)		718/8022 (9.0)	591/6675 (8.9)	127/1347 (9.4)	0.501
Physical activity, n (%)	Low	1415/7996 (17.7)	1228/6651 (18.5)	187/1345 (13.9)	< 0.001
	Medium	4645/7996 (58.1)	3891/6651 (58.5)	754/1345 (56.1)	
	High	1936/7996 (24.2)	1532/6651 (23.0)	404/1345 (30.0)	
SBP, mmHg		121.8 ± 18.84	122.8 ± 18.77	117.3 ± 18.52	< 0.001
DBP, mmHg		80.8 ± 10.82	81.1 ± 10.87	79.1 ± 10.44	< 0.001
MAP, mmHg		94.5 ± 12.37	95.0 ± 12.37	91.9 ± 12.07	< 0.001
FPG, mg/dL		97.0 [90.0, 107.0]	98.0 [91.0, 109.0]	94.0 [88.0, 100.0]	< 0.001
Creatinine, mg/dL		0.80 [0.70, 0.94]	0.80 [0.70, 0.95]	0.79 [0.69, 0.91]	< 0.001
Total cholesterol, mg/dL		170.0 [145.0, 197.0]	173.0 [144.0, 203.0]	162.0 [146.0, 177.0]	< 0.001
LDL-C, mg/dL		111.0 [90.0, 134.0]	115.0 [90.0, 139.0]	102.0 [86.0, 115.0]	< 0.001
HDL-C, mg/dL		43.0 [36.0, 52.0]	41.0 [35.0, 49.0]	53.0 [47.0, 60.0]	< 0.001
Triglyceride, mg/dL		124.0 [88.0, 175.0]	139.0 [98.0, 188.0]	80.0 [63.0, 103.0]	< 0.001

BMI, body mass index; CAD, coronary artery disease; DBP, diastolic blood pressure; HDL-C, high-density lipoprotein-cholesterol; LDL-C: low-density lipoprotein-cholesterol; MAP, mean arterial pressure; SBP, systolic blood pressure. Categorical variables were presented as numbers (percentages in the column). Numerical variables were presented as mean ± standard deviation in a normally distributed variable and median [interquartile range] in non-parametric data. ^a^Dyslipidemia was defined based on laboratory findings, previous diagnosis, or self-reported use of lipid-lowering medications. ^b^*P* values < 0.05 were statistically significant.

###  Prevalence

 In this study, the overall prevalence of dyslipidemia was 83.2%. The most common lipid abnormality was low HDL-C, with a prevalence of 55.1%, followed by hypertriglyceridemia at 36.4%, high LDL-C at 23.1%, and hypercholesterolemia at 23.1%, respectively. The age-sex standardized prevalence of dyslipidemia was estimated to be 82.7% (95% CI: 80.1%-85.0%) in Tehran. The age-sex standardized prevalence for each lipid abnormality is presented in (Table 2). We found a higher prevalence of dyslipidemia, hypercholesterolemia, high LDL-C, and low HDL-C in women; however, hypertriglyceridemia and high LDL/HDL ratio were significantly more common in men (Table 3 and Table 4). The mean age was considerably higher in those with hypercholesterolemia, while low HDL-C and high LDL/HDL ratio were more frequent at a younger age. We observed no age differences in participants with high LDL-C or hypertriglyceridemia.

**Table 2:** Age-sex Standardized Prevalence of Dyslipidemia and Abnormal Lipid Biomarkers in Tehran

	**Dyslipidemia**^a^	**Hypertriglyceridemia**^b^	**Hypercholesterolemia**^b^	**High LDL-C**^b^	**Low HDL-C**^b^	**High LDL/HDL**^b^
Total (%)	82.7 (80.1, 85.0)	36.9 (33.8, 40.1)	22.5 (19.9, 25.4)	29.0 (26.1, 32.1)	55.9 (52.6, 59.2)	54.1 (50.9, 57.3)
Women (%)	82.1 (79.7, 84.3)	31.6 (28.7, 34.6)	25.8 (23.1, 28.7)	30.5 (27.7, 33.5)	56.5 (53.2, 59.6)	43.2 (40.0, 46.4)
Men (%)	83.3 (80.6, 85.8)	42.4 (39.1, 45.8)	19.1 (16.6, 22)	27.4 (24.5, 30.6)	55.3 (51.9, 58.7)	65.3 (62.0, 68.5)

HDL-C, high-density lipoprotein-cholesterol; LDL-C, low-density lipoprotein-cholesterol. The age-sex standardized prevalence was reported as a percentage (95% confidence interval). ^a^Dyslipidemia was defined based on laboratory findings, previous diagnosis, or self-reported use of lipid-lowering medications. ^b^Hypercholesterolemia was defined as total cholesterol ≥ 200 mg/dL (≥ 5.2 mmol/L), high LDL-C defined as LDL-C ≥ 130 mg/dL (≥ 3.4 mmol/L), low HDL-C defined as HDL-C < 40 mg/dL (< 1.03 mmol/L) in men, and < 50 mg/dL (< 1.29 mmol/L) in women, hypertriglyceridemia was defined as triglyceride ≥ 150 mg/dL (≥ 1.7 mmol/L), and high LDL/HDL as ratio > 2.5.

**Table 3:** Comparison of the Participants’ Characteristics Within Abnormal Triglyceride and Total Cholesterol Subgroups in the Cohort Population Irrespective of Dyslipidemia History

		**Triglyceride**		***P*** **Value**^b^	**Total Cholesterol**		***P*** **Value**^b^
		**High**^a^ ** (N=2892 [36.4%])**	**Normal (N=5058 [63.6%])**		**High**^a^ **(N=1837 [23.1%])**	**Normal** **(N=6113 [76.9%])**	
Age, year		53.5 ± 11.7	53.8 ± 13.2	0.310	54.5 ± 11.6	53.5 ± 13.0	0.001
Gender, n (%)	Women	1397 (48.3)	2909 (57.5)	< 0.001	1149 (62.5)	3157 (51.6)	< 0.001
	Men	1495 (51.7)	2149 (42.5)		688 (37.5)	2956 (48.4)	
BMI, kg/m^2^		29.1 ± 4.6	27.4 ± 4.8	< 0.001	28.4 ± 4.6	27.9 ± 4.9	< 0.001
BMI subgroups, n (%)	< 20	15 (0.5)	203 (4.1)	< 0.001	27 (1.5)	191 (3.2)	< 0.001
	20-24.9	509 (17.7)	1473 (29.5)		404 (22.3)	1578 (26.0)	
	25-29.9	1288 (44.8)	2009 (40.2)		783 (43.3)	2514 (41.5)	
	30-34.5	764 (26.6)	958 (19.2)		443 (24.5)	1279 (21.1)	
	≥ 35	296 (10.3)	357 (7.1)		153 (8.5)	500 (8.2)	
Waist-to-hip ratio		0.93 ± 0.07	0.91 ± 0.07	< 0.001	0.92 ± 0.07	0.91 ± 0.07	0.121
Education years, n (%)	Illiterate	212 (7.4)	341 (6.8)	0.501	132 (7.2)	421 (6.9)	0.817
	1-5	287 (10.0)	515 (10.2)		187 (10.2)	615 (10.1)	
	6-12	1518 (52.6)	2613 (51.8)		952 (52.2)	3179 (52.1)	
	> 12	867 (30.1)	1574 (31.2)		554 (30.4)	1887 (30.9)	
Diabetes mellitus, n (%)		678 (23.4)	775 (15.3)	< 0.001	276 (15.0)	1177 (19.3)	< 0.001
Hypertension, n (%)		916 (31.7)	1307 (25.9)	< 0.001	468 (25.6)	1755 (28.8)	0.009
Coronary heart disease, n (%)		272 (9.4)	462 (9.2)	0.696	97 (5.3)	637 (10.4)	< 0.001
Cerebrovascular disease, n (%)		40 (1.4)	69 (1.4)	0.948	18 (1.0)	91 (1.5)	0.104
Chronic kidney disease, n (%)		29 (1.0)	41 (0.8)	0.378	13 (0.7)	57 (0.9)	0.366
Statin, n (%)		702 (24.3)	979 (19.4)	< 0.001	236 (12.9)	1445 (23.7)	< 0.001
Non-statin lipid-lowering agents, n (%)		101 (3.5)	53 (1.0)	< 0.001	43 (2.3)	111 (1.8)	0.145
Family history of CAD, n (%)		281 (9.7)	470 (9.3)	0.534	183 (10.0)	568 (9.3)	0.389
Tobacco, n (%)	Current	598 (20.7)	934 (18.5)	0.008	334 (18.3)	1198 (19.6)	0.102
	Former	129 (4.5)	186 (3.7)		61 (3.3)	254 (4.2)	
	Never	2158 (74.8)	3922 (77.8)		1431 (78.4)	4649 (76.2)	
Alcohol, n (%)		310 (10.8)	401 (8.0)	< 0.001	186 (10.2)	525 (8.6)	0.040
Physical activity, n (%)	Low	533 (18.6)	851 (17.0)	0.001	336 (18.5)	1048 (17.3)	0.238
	Medium	1696 (59.3)	2877 (57.4)		1024 (56.4)	3549 (58.6)	
	High	629 (22.0)	1284 (25.6)		455 (25.1)	1458 (24.1)	
SBP, mmHg		124.6 ± 18.6	120.1 ± 18.8	< 0.001	124.1 ± 19.2	121.1 ± 18.6	< 0.001
DBP, mmHg		82.3 ± 10.8	79.9 ± 10.7	< 0.001	82.9 ± 11.1	80.1 ± 10.7	< 0.001
MAP, mmHg		96.4 ± 12.3	93.3 ± 12.2	< 0.001	96.6 ± 12.7	93.8 ± 12.2	< 0.001
FPG, mg/dL		100.0 [93.0, 114.0]	96.0 [89.0, 104.0]	< 0.001	98.0 [92.0, 108.0]	97.0 [90.0, 107.0]	< 0.001
Creatinine, mg/dL		0.83 [0.71, 0.97]	0.80 [0.69, 0.92]	< 0.001	0.80 [0.70, 0.93]	0.80 [0.70, 0.94]	0.765

BMI, body mass index; CAD: coronary artery disease; DBP, diastolic blood pressure; MAP, mean arterial pressure; SBP, systolic blood pressure. Categorical variables were presented as numbers (percentages in the column). Numerical variables were presented as mean ± standard deviation in normally distributed variables and median [interquartile range] in non-parametric data. ^a^High triglyceride was defined as triglyceride ≥ 150 mg/dL (≥ 1.7 mmol/L) and high total cholesterol was defined as total cholesterol ≥ 200 mg/dL (≥ 5.2 mmol/L). ^b^*P* values < 0.05 were statistically significant.

**Table 4:** Comparison of the Participants’ Characteristics within Abnormal LDL-C, HDL-C, and LDL/HDL Ratio Subgroups in Cohort Population Irrespective of Dyslipidemia History

		**LDL-C**^a^		***P*** **Value**^b^	**HDL-C**		***P*** **Value**^b^	**LDL/HDL Ratio**		***P*** **Value**^b^
		**High**^a^ **(N=2332 [29.3%])**	**Normal (N=5618 [70.7%])**		**Low**^a^ **(N=4383 [55.1%])**	**Normal (N=3567 [44.9%])**		**High**^a^ ** (N=4174 [52.5%])**	**Normal (N=3776 [47.5%])**	
Age, year		53.9 ± 11.7	53.6 ± 13.1	0.339	52.7 ± 12.4	54.9 ± 12.9	< 0.001	52.8 ± 12.1	54.7 ± 13.3	< 0.001
Gender, n (%)	Women	1349 (57.8)	2957 (52.6)	< 0.001	2419 (55.2)	1887 (52.9)	0.042	1871 (44.8)	2435 (64.5)	< 0.001
	Men	983 (42.2)	2661 (47.4)		1964 (44.8)	1680 (47.1)		2303 (55.2)	1341 (35.5)	
BMI, kg/m^2^		28.3 ± 4.7	27.8 ± 4.9	< 0.001	28.6 ± 4.8	27.2 ± 4.7	< 0.001	28.4 ± 4.6	27.5 ± 5.0	< 0.001
BMI subgroups, n (%)	< 20	38 (1.7)	180 (3.2)	< 0.001	53 (1.2)	165 (4.7)	< 0.001	59 (1.4)	159 (4.3)	< 0.001
	20-24.9	525 (22.8)	1457 (26.2)		926 (21.3)	1056 (29.9)		889 (21.5)	1093 (29.2)	
	25-29.9	1021 (44.4)	2276 (40.9)		1872 (43.1)	1425 (40.4)		1839 (44.5)	1458 (39.0)	
	30-34.5	520 (22.6)	1202 (21.6)		1064 (24.5)	658 (18.7)		989 (23.9)	733 (19.6)	
	≥ 35	198 (8.6)	455 (8.2)		431 (9.9)	222 (6.3)		359 (8.7)	294 (7.9)	
Waist-to-hip ratio		0.92 ± 0.07	0.91 ± 0.07	0.013	0.92 ± 0.07	0.91 ± 0.08	< 0.001	0.93 ± 0.07	0.90 ± 0.08	< 0.001
Education years, n (%)	Illiterate	156 (6.7)	397 (7.1)	0.179	340 (7.8)	213 (6.0)	0.022	274 (6.6)	279 (7.4)	0.524
	1-5	238 (10.2)	564 (10.1)		488 (11.1)	314 (8.8)		419 (10.1)	383 (10.2)	
	6-12	1214 (52.3)	2917 (52.0)		2293 (52.4)	1838 (51.8)		2186 (52.5)	1945 (51.6)	
	> 12	714 (30.7)	1727 (30.8)		1256 (28.7)	1185 (33.4)		1281 (30.8)	1160 (30.8)	
Diabetes mellitus, n (%)		320 (13.7)	1133 (20.2)	< 0.001	891 (20.3)	562 (15.8)	< 0.001	650 (15.6)	803 (21.3)	< 0.001
Hypertension, n (%)		555 (23.9)	1668 (29.7)	< 0.001	1295 (29.6)	928 (26.1)	0.001	1019 (24.5)	1204 (31.9)	< 0.001
Coronary heart disease, n (%)		123 (5.3)	611 (10.9)	< 0.001	422 (9.6)	312 (8.8)	0.191	264 (6.3)	470 (12.5)	< 0.001
Cerebrovascular disease, n (%)		23 (1.0)	86 (1.5)	0.059	70 (1.6)	39 (1.1)	0.057	50 (1.2)	59 (1.6)	0.164
Chronic kidney disease, n (%)		13 (0.6)	57 (1.0)	0.047	49 (1.1)	21 (0.6)	0.012	33 (0.8)	37 (1.0)	0.367
Statin, n (%)		236 (10.2)	1445 (25.8)	< 0.001	952 (21.8)	729 (20.5)	0.188	495 (11.9)	1186 (31.5)	< 0.001
Non-statin lipid-lowering agents, n (%)		42 (1.8)	112 (2.0)	0.578	113 (2.6)	41 (1.1)	< 0.001	87 (2.1)	67 (1.8)	0.314
Family history of CAD, n (%)		226 (9.7)	525 (9.3)	0.631	451 (10.3)	300 (8.4)	0.004	386 (9.2)	365 (9.7)	0.524
Tobacco, n (%)	Current	431 (18.6)	1101 (19.6)	0.064	857 (19.6)	675 (19.0)	0.203	948 (22.8)	584 (15.5)	< 0.001
	Former	77 (3.3)	238 (4.2)		159 (3.6)	156 (4.4)		159 (3.8)	156 (4.1)	
	Never	1815 (78.1)	4265 (76.1)		3360 (76.8)	2720 (76.6)		3053 (73.4)	3027 (80.4)	
Alcohol, n (%)		213 (9.2)	498 (8.9)	0.689	348 (8.0)	363 (10.3)	< 0.001	424 (10.2)	287 (7.7)	< 0.001
Physical activity, n (%)	Low	397 (17.2)	987 (17.8)	0.126	833 (19.2)	551 (15.6)	< 0.001	708 (17.1)	676 (18.1)	0.515
	Medium	1317 (57.0)	3256 (58.6)		2586 (59.5)	1987 (56.4)		2420 (58.6)	2153 (57.6)	
	High	597 (25.8)	1316 (23.7)		925 (21.3)	988 (28.0)		1003 (24.3)	910 (24.3)	
SBP, mm Hg		123.5 ± 19.0	121.0 ± 18.7	< 0.001	121.9 ± 18.6	121.5 ± 19.1	0.328	123.1 ± 18.5	120.2 ± 19.0	< 0.001
DBP, mm Hg		82.3 ± 11.0	80.2 ± 10.7	< 0.001	80.9 ± 10.8	80.6 ± 10.8	0.170	81.7 ± 10.9	79.7 ± 10.6	< 0.001
MAP, mm Hg		96.0 ± 12.6	93.8 ± 12.2	< 0.001	94.6 ± 12.2	94.2 ± 12.5	0.202	95.5 ± 12.4	93.2 ± 12.2	< 0.001
FPG, mg/dL		98.0 [92.0, 107.0]	97.0 [90.0, 107.0]	< 0.001	98.0 [90.0, 109.0]	96.0 [91.0, 105.0]	< 0.001	98.0 [92.0, 107.0]	96.0 [89.0, 106.0]	< 0.001
Creatinine, mg/dL		0.81 [0.70, 0.94]	0.80 [0.70, 0.94]	0.023	0.80 [0.69, 0.93]	0.81 [0.70, 0.95]	< 0.001	0.83 [0.71, 0.96]	0.78 [0.68, 0.90]	< 0.001

BMI, body mass index; CAD: coronary artery disease; DBP, diastolic blood pressure; HDL-C, high-density lipoprotein-cholesterol; LDL-C, low-density lipoprotein-cholesterol; MAP, mean arterial pressure; SBP, systolic blood pressure. ^a^High LDL-C defined as LDL-C ≥ 130 mg/dL (≥ 3.4 mmol/L), low HDL-C defined as HDL-C < 40 mg/dL (< 1.03 mmol/L) in men, and < 50 mg/dL (< 1.29 mmol/L) in women, and high LDL/HDL as ratio > 2.5. ^b^*P* values < 0.05 were statistically significant. Categorical variables were presented as numbers (percentages in the column). Numerical variables were presented as mean ± standard deviation in normally distributed variables and median [interquartile range] in non-parametric data.

###  Dyslipidemia and Abnormal Lipid Profile

 We found that individuals with dyslipidemia were more likely to be older, overweight (BMI ≥ 25; 75.6% vs. 54.5%, *P* value < 0.001), or obese (BMI ≥ 30; 32.5% vs. 19.7%, *P* value < 0.001) compared to those without dyslipidemia. Additionally, dyslipidemia was associated with lower physical activity, higher waist-to-hip ratio, and all comorbidities including diabetes mellitus, hypertension, CAD, cerebrovascular disease, and chronic kidney disease. Moreover, these individuals had higher SBP, DBP, MAP, FPG, and creatinine levels during their first visit compared to those without dyslipidemia. Nevertheless, a family history of CAD did not influence the frequency of dyslipidemia (*P* value: 0.341) (Table 1).

 After excluding 122 patients due to non-fasting laboratory test results, we compared the baseline characteristics of entire cohort participants within the abnormal lipid profile subgroups irrespective of dyslipidemia history (Table 3 and Table 4). Our results demonstrated that high BMI was associated with a higher prevalence of all lipid abnormalities. In addition, individuals with a higher waist-to-hip ratio were more likely to have abnormal lipid profiles except for hypercholesterolemia. We found that patients with diabetes, hypertension, or CAD had lower TC, LDL-C, and LDL/HDL ratios; however, they had a higher frequency of low HDL-C and hypertriglyceridemia. In terms of smoking and drinking habits, not having a history of tobacco smoking was associated with a considerably lower triglyceride level. Participants with a history of alcohol consumption were found to have a greater prevalence of hypercholesterolemia, and triglyceridemia, with higher HDL-C levels and LDL/HDL ratio.

###  Age-Sex Disparities

**Figure 1:**
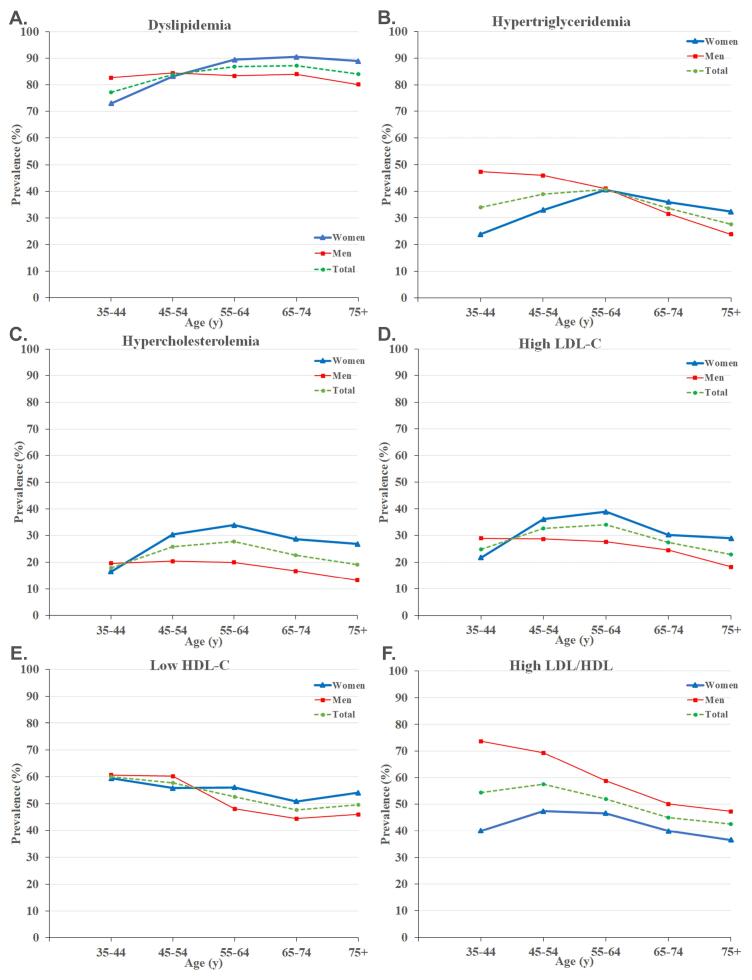
Prevalence of Dyslipidemia (A), Hypertriglyceridemia (B), Hypercholesterolemia(C), High LDL-C (D), Low HDL-C (E), and High LDL/HDL Ratio (F) Stratified by Age and Sex. HDL-C, high-density lipoprotein cholesterol; LDL-C, low-density lipoprotein cholesterol

 The prevalence of dyslipidemia and abnormal lipid profiles were evaluated in both men and women in five different age groups (Figure 1, Table S1). There was an increasing pattern for dyslipidemia with aging followed by a decrease in the ‘ + 75 years’ group (Figure 1A). Furthermore, the prevalence of hypertriglyceridemia, hypercholesterolemia, and high LDL-C increased with aging, peaked in the ‘55-64 years’ group, and decreased afterward (Figure 1B-D).

 Regarding sex differences, men had a considerably higher prevalence of dyslipidemia among individuals aged 35-44. Moreover, dyslipidemia showed a rising trend in women as they aged, with women being more prone to dyslipidemia after the age of 55 compared to men (Figure 1A). We observed similar increasing patterns for hypercholesterolemia and high LDL-C, peaking in the ‘55-64 years’ group. Women had a significantly higher prevalence of hypercholesterolemia and high LDL-C in individuals aged ≥ 45 years (Figure 1C-D). It was found that hypertriglyceridemia was significantly more common in men < 55 years and low HDL-C in men aged 45-54 years (Figure 1B and Figure 1E). Eventually, significantly higher LDL/HDL ratios were observed in men across all ages, with the highest difference of 33.6% in the ‘35-44 years’ group (Figure 1F, Table S1).

###  Geographical Distribution

**Figure 2:**
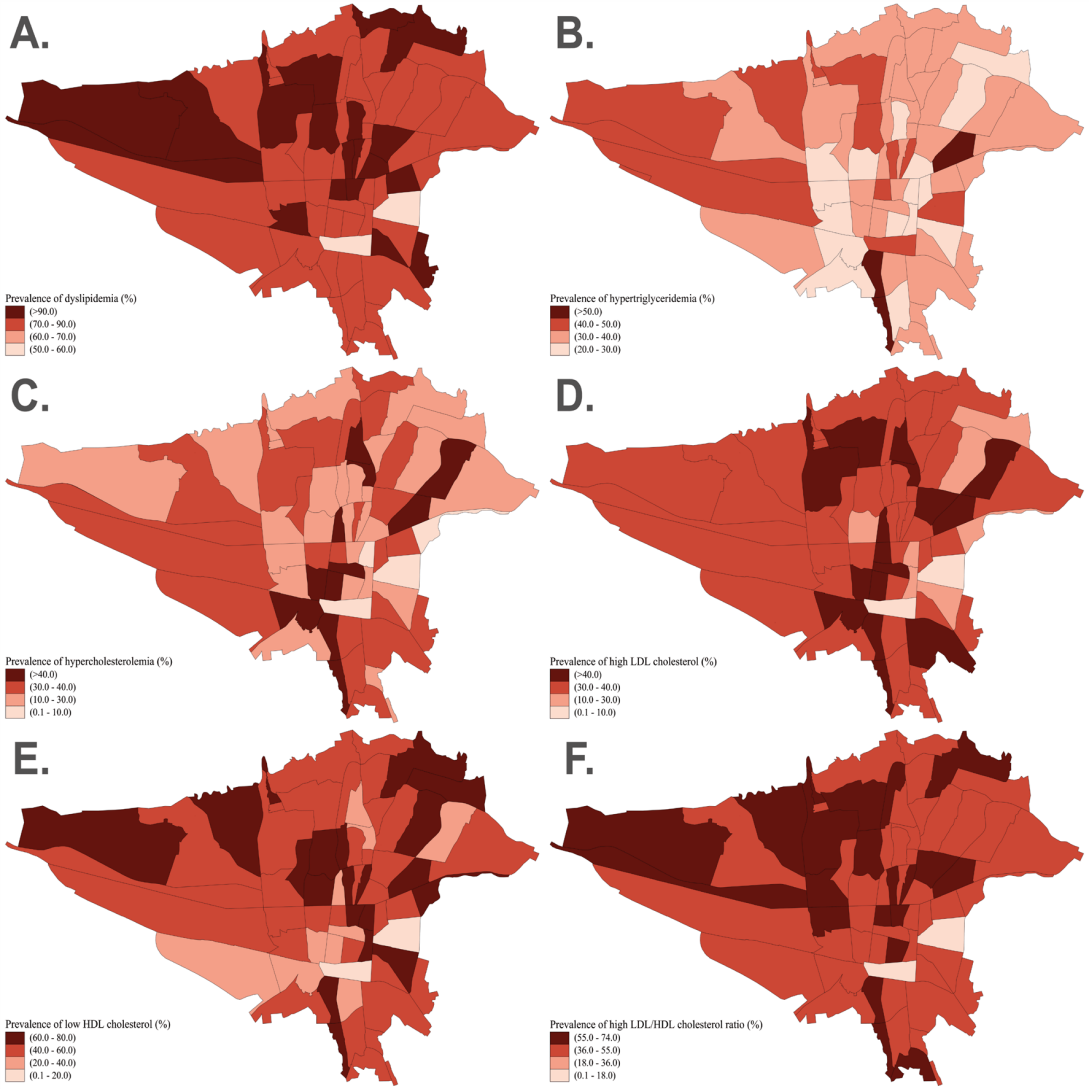
Geographic distribution of dyslipidemia (A), hypertriglyceridemia (B), hypercholesterolemia (C), high LDL-C (D), low HDL-C (E), and high LDL/HDL ratio (F) based on the zip code districts of Tehran. HDL-C, high-density lipoprotein cholesterol; LDL-C, low-density lipoprotein cholesterol

 The geographical distribution of our findings based on zip code districts of Tehran illustrates that the prevalence of dyslipidemia, low HDL-C, and high LDL/HDL ratio was higher in the northern half of Tehran (Figure 2A, E, F). In addition, hypercholesterolemia was higher in the southern half, and high LDL-C was more prevalent in the middle-northern and southern regions of Tehran (Figure 2C, D). However, we observed no specific distributional pattern for hypertriglyceridemia in different areas (Figure 2B).

## Discussion

 This study estimated the prevalence of dyslipidemia and its subgroups among the adult population of Tehran aged ≥ 35 years using the TeCS recruitment data. The age-sex standardized prevalence of dyslipidemia was 82.7%. We found low HDL-C as the most common lipid abnormality followed by hypertriglyceridemia, high LDL-C, and hypercholesterolemia, respectively. Moreover, more than half of the Tehran adult population was estimated to have a high LDL/HDL ratio.

 The WHO STEPS national survey results in 2021 concluded that 81.0% of the Iranian population (aged ≥ 25 years) had dyslipidemia.^8^ Their finding indicated that low HDL-C (68.4%) was the most prevalent lipid abnormality, followed by hypertriglyceridemia (39.7%), hypercholesterolemia (21.2%), and high LDL-C (16.4%). Meanwhile, a meta-analysis of population-based studies from 1994 to 2015 estimated the prevalence of hypercholesterolemia at 42%, high LDL-C at 40%, and low HDL-C at 42%.^9^ A comparison among these studies, considering the same cut-offs, reveals a decreasing pattern in hypercholesterolemia and high LDL-C prevalence; however, there is an increasing trend in low HDL-C levels during the same period. The Tehran metropolis is the most populous city in Iran with great ethnic diversity, which has experienced rapid urbanization in the past decades.^21^ Therefore, Tehran has a higher prevalence of dyslipidemia which can be possibly attributed to air pollution, a sedentary lifestyle, and unhealthy dietary habits compared to less populated provinces such as Golestan (68.5%), Kerman (74.1%), Mazandaran (76.6%), and Semnan (76.8%).^8^ However, more studies are warranted to find evidence between the following risk factors and dyslipidemia.^22,23^ A previous study has demonstrated a favorable trend in reducing TC and non-HDL-C from 1999 to 2011 in Tehran; however, it was limited due to the small sample size and single-district sampling.^24^ Since then, no other study has evaluated the prevalence of dyslipidemia in Tehran, and its current net prevalence remains unknown. Therefore, our results could provide a better image of dyslipidemia prevalence and its changing patterns among the adult population of Tehran by the upcoming follow-up phases of TeCS.

 Our results showed that one out of three adult residents of Tehran has hypertriglyceridemia, which is higher than the rest of the country.^8,25^ Nonetheless, the latest WHO STEPS survey showed a high prevalence of hypertriglyceridemia in men who live in Tehran, while the prevalence was higher in women who lived in other cities.^8^ Similarly, we have observed a significantly higher prevalence of hypertriglyceridemia in men; this difference has more than doubled in individuals aged 35-44 and decreased with advancing age. Previous studies have reported a steady trend in the prevalence of hypertriglyceridemia from 1999 to 2011 in Tehran, but now it has diminished by 9.6% since 2011, considering the same cut-off value.^24-26^ In addition, some risk factors, such as higher BMI, diabetes, hypertension, tobacco smoking, and alcohol consumption, were associated with an increased risk of hypertriglyceridemia.

 This study indicated a significantly greater prevalence of hypercholesterolemia and high LDL-C among women than men, which is similar to findings from previous studies conducted in other provinces in Iran.^8,9^ Moreover, hypercholesterolemia and high LDL-C prevalence increased in the middle-aged, peaked among individuals aged 55-64 years, and decreased afterward with the same pattern in both genders. Previous studies have reported similar levels of TC and LDL-C between men and women during the first two decades of life, with a trend of increase in both sexes with advancing age.^18,25^ Of note, women experienced a steeper rise in TC and LDL-C levels compared to men. One explanation can be lower estrogen levels in women over 50 years, leading to increased lipid abnormalities after menopause.^27^ We observed an increasing pattern regarding the prevalence of hypercholesterolemia and high LDL-C with aging, which peaks in the ‘55-64’ age group. It emphasizes that during the fourth and fifth decades of life, individuals are at greater risk for developing hypercholesterolemia and high LDL-C by aging. Thus, implementing public health strategies to increase health literacy and developing screening programs can help minimize the burden of CVDs, especially in this age group.^28^

 After evaluating the geographical distribution of lipid parameters, we observed a higher prevalence of dyslipidemia, low HDL-C, and high LDL/HDL ratio in the northern regions of Tehran (traditionally known as higher socio-economic status region), while hypercholesterolemia and high LDL-C were more prevalent in the southern regions. We hypothesize that these patterns may be attributed to higher socio-economic status and more sedentary lifestyles in the northern districts, and to greater air pollution exposure and unhealthy dietary habits in the southern regions of Tehran.^7,17^ Nevertheless, these associations warrant further socio-economic studies to elucidate the underlying factors contributing to the observed geographical disparities in lipid profiles.

 The current age-sex standardized prevalence of hypercholesterolemia and high LDL-C in Tehran was lower than in many countries, such as the United States, China, Poland, and Turkey, using the same cut-off values.^29-32^ We believe that differences in lifestyles, dietary habits, genetic factors, and time of studies can cause this heterogeneity among different societies. Nevertheless, there was a 12.1% and 17.5% reduction in mean TC and LDL-C levels since 2011.^24^ National policies established in 2000 may have contributed to this favorable trend. These policies restricted using trans fatty acids in all oil products and tried to raise public awareness regarding the risks of dietary intake of saturated fats.^33^ Besides, the observed decrease in dyslipidemia may have been explained not only by positive lifestyle changes but also by the increased utilization of lipid-lowering medications.^34^ Nevertheless, we must emphasize that high LDL-C was still related to 16.1% (95% uncertainty interval (UI): 12.2%–20.5%) of deaths and 7.8% (95% UI: 6.2–9.7) of disability-adjusted life years of non-communicable diseases in 2019 among the Iranian population.^1^

 The high prevalence of dyslipidemia was mainly attributed to the high prevalence of low HDL-C among residents of Tehran, which is one of the highest reported values worldwide.^29-32^ We observed that women were more prone to have low HDL-C, but during the fourth and fifth decades of life, low HDL-C was more prevalent in men. According to global physical activity reports, Iran suffers from a high prevalence of inappropriate physical activity.^35^ Similarly, we noticed a greater prevalence of low HDL-C in those with considerably inadequate physical activity within the adult population. Iranians’ dietary habits, which are high in carbohydrates, may also contribute to HDL-C abnormality. Replacing saturated fatty acids with carbohydrates was associated with no change in TC and LDL-C levels but also an unfavorable decrease in HDL-C and an increase in triglyceride.^36^ On the other hand, the prohibition of alcohol use and its low prevalence in society may be another reason for the high prevalence of low HDL-C in Tehran. It should be noted that the real estimation of alcohol consumption could be different from the values reported in this study, as there is a lack of information or underreporting by patients who know that alcohol consumption is prohibited.

 New evidence suggests the LDL-C/HDL-C ratio as a novel marker associated with coronary atherosclerosis progression, myocardial infarction, and even adverse outcomes within one year in patients undergoing coronary angioplasty.^37,38^ It was proposed as a significant predictor for coronary atherosclerotic heart disease, as it takes into account both LDL-C and HDL-C levels simultaneously, with 64.5% sensitivity and 61.3% specificity for a cut-off value of > 2.5.^19^ We observed a significant prevalence of a high LDL-C/HDL-C ratio (52.5%), with a 20% higher prevalence among men than women. This between-gender difference was more significant among the ‘35-44’ and ‘45-54’ age groups and decreased with aging. We must highlight that the prevalence of high LDL-C/HDL-C was more than 1.5 times higher in men aged 35-44 compared to women.

 Despite several strengths of this study, including its comprehensive, randomized sample size that investigated the prevalence of dyslipidemia and lipid abnormalities across all geographical districts of Tehran, it has some limitations. First, it is a cross-sectional study, which inherits certain biases. Second, the study’s enrollment was limited to individuals aged 35 years and above in TeCS, limiting the representativeness of our results for the general population due to the absence of data on younger individuals. Third, although standardized procedures were employed for lipid measurements, the inherent biological variability and reliance on self-reported data could potentially introduce measurement and information biases. Efforts were made to minimize these through validated methodologies and the cross-checking of medical records. Fourth, despite adjusting for known confounders such as lifestyle factors and comorbidities, residual confounding by unmeasured or unknown factors cannot be entirely ruled out. Acknowledging these limitations is crucial when interpreting the results, as it emphasizes the need for further research employing diverse designs to more comprehensively explore dyslipidemia.

## Conclusion

 We found that four out of five adult residents (aged ≥ 35 years) of Tehran have dyslipidemia, with low HDL-C being the most prevalent lipid abnormality. In addition, a high LDL/HDL ratio was observed in more than half of the study population. This dyslipidemia profiling on a large-scale random sample from Tehran provides valuable insights for healthcare policymakers aiming to develop and implement comprehensive preventative measures to decrease the incidence of dyslipidemia-related morbidity and mortality. Additionally, it is crucial to focus future research on high-risk populations to assess the efficacy of various prevention and treatment programs in improving health literacy, promoting medication adherence, and encouraging healthy lifestyle behaviors.

## Supplementary Files

Supplementary file 1 contains Table S1.
