# Supplementary material for: Epidemiology and Prevalence of Dyslipidemia Among Adult Population of Tehran: The Tehran Cohort Study
Source: Arch Iran Med. 2024 Feb 1;27(2):51–61. doi: 10.34172/aim.2024.10 (PMC11017263; doi:10.34172/aim.2024.10)
Supplement: Supplementary file 1 — contains Table S1. [file aim-27-51-s001.pdf]

Arch Iran Med. February 2024;27(2):51-S1

10.34172/aim.2024.10

Supplementary file 1

Table S1. Prevalence of Dyslipidemia and Abnormal Lipid Profile within Age-sex Subgroups.

	Dyslipidemia*				Hypertriglyceridemia#				Hypercholesterolemia#				High LDL-C#				Low HDL-C#				High LDL/HDL#			
Age (year)	Total	Women	Men	P**	Total	Women	Men	P**	Total	Women	Men	P**	Total	Women	Men	P**	Total	Women	Men	P**	Total	Women	Men	P
35-44	77.20%	73.08%	82.69%	<0.001	33.88%	23.84%	47.35%	<0.001	17.83%	16.54%	19.56%	0.064	24.81%	21.74%	28.93%	<0.001	59.94%	59.39%	60.67%	0.543	54.42%	40.06%	73.67%	<0.001
45-54	83.81%	83.28%	84.45%	0.461	38.90%	32.96%	45.94%	<0.001	25.78%	30.36%	20.35%	<0.001	32.69%	36.08%	28.67%	<0.001	57.81%	55.77%	60.23%	0.038	57.48%	47.44%	69.37%	<0.001
55-64	86.84%	89.46%	83.43%	<0.001	40.68%	40.49%	40.93%	0.849	27.83%	33.90%	19.98%	<0.001	33.99%	38.89%	27.65%	<0.001	52.58%	56.03%	48.11%	0.001	51.94%	46.61%	58.83%	<0.001
65-74	87.20%	90.48%	84.02%	0.001	33.68%	35.92%	31.52%	0.114	22.60%	28.70%	16.70%	<0.001	27.36%	30.28%	24.53%	0.028	47.62%	50.88%	44.46%	0.029	45.11%	39.96%	50.09%	0.001
75+	84.06%	88.93%	80.19%	0.005	27.56%	32.34%	23.84%	0.029	19.18%	26.81%	13.25%	<0.001	22.91%	28.94%	18.21%	0.003	49.53%	54.04%	46.03%	0.065	42.64%	36.60%	47.35%	0.012

HDL-C, high-density lipoprotein-cholesterol; LDL-C, low-density lipoprotein-cholesterol.

*Dyslipidemia was defined based on laboratory findings, previous diagnosis, or self-reported use of lipid-lowering medications.

#Hypercholesterolemia was defined as total cholesterol ≥200 mg/dL (≥5.2 mmol/L), high LDL-C defined as LDL-C ≥130 mg/dL (≥3.4 mmol/L), low HDL-C defined as HDL-C <40 mg/dL (<1.03 mmol/L) in men, and <50 mg/dL (<1.29 mmol/L) in women, hypertriglyceridemia was defined as triglyceride≥150 mg/dL (≥1.7 mmol/L), and high LDL/HDL as ratio >2.5.

***P* values <0.05 were statistically significant.
